# Everyone should get to know that it’s okay to feel bad: adolescents’ and parents’ experiences of participating in a preventive emotion regulation skills training in a Swedish school setting — a qualitative study

**DOI:** 10.1080/17482631.2026.2679416

**Published:** 2026-05-23

**Authors:** Andersson H., Aspeqvist E., Korhonen L., Gustafsson B. M., Zetterqvist M.

**Affiliations:** a Center for Social and Affective Neuroscience, Department of Biomedical and Clinical Sciences, Linköping University, Linköping, Sweden; b Clinical Department of Child and Adolescent Psychiatry in Linköping, Region Östergötland, Linköping, Sweden; c Barnafrid, Swedish National Center on Violence Against Children, Department of Biomedical and Clinical Sciences, Linköping University, Linköping, Sweden; d Psychiatric Clinic, Högland Hospital, Department of Psychiatry and Rehabilitation, Region Jönköping, Sweden; e CHILD research environment, Jönköping University, Jönköping, Sweden

**Keywords:** Adolescents, emotion regulation, parents, prevention, school, skills training

## Abstract

**Background:**

Difficulties in emotion regulation (ER) have been identified as a core mechanism in mental health problems. Preventive interventions targeting ER in adolescents, both in universal and selected settings, show promising effects in reducing ER difficulties and supporting general health, though further research is needed to confirm these findings. Earlier studies also highlight the potential value of involving parents in such interventions to strengthen parent–child relationships and promote healthy ER, but this approach also requires further exploration. The aim of the current study was to explore how adolescents and parents described their experiences and perceptions of participating jointly in a preventive ER skills training in a school setting. Such experiential and perception-focused insights underscore the need for a qualitative approach.

**Methods:**

The skills training consisted of five sessions of psychoeducation about emotions and ER skills. Twelve participants (seven parents and five adolescents) were interviewed. The interviews were transcribed and analyzed with reflexive thematic analysis.

**Results:**

The participants expressed a general need for emotional awareness, including understanding emotions and ER, as well as recognizing the role of emotions in everyday issues. The perceived stigma associated with mental health and emotional expression, along with a lack of knowledge about emotions, was seen as concealing the need for emotional awareness. Parents were viewed as facilitating the learning and application of ER skills among adolescents. Participants experienced insights (e.g., increased emotional awareness) that benefited their daily lives, but also perceived challenges with preventive ER skills training regarding timing and the need for continued practice to maintain skills. Participants found the ER skills training to be valuable and meaningful.

**Conclusion:**

The results highlight both the benefits and challenges of jointly targeting ER preventively in adolescents and their parents within a school setting, particularly with regard to recruitment and evaluation. The findings also contribute to ongoing discussions in prevention research about the optimal timing of interventions, ER focus, and parental involvement.

## Introduction

Mental health problems typically have their onset during adolescence, with an estimated prevalence of approximately 14% for mental disorders in 10–19-year-olds (WHO, 2021). The COVID-19 pandemic has also shown to negatively impact adolescent stress and mental health (Pieh et al., 2022), with a potential increase in depressive and anxiety symptoms (Racine et al., 2021). It has furthermore been associated with potential increases in nonsuicidal self-injury (NSSI) and suicidal behaviours (Garcia-Fernandez et al., 2023; Zetterqvist et al., 2021). Difficulties in emotion regulation (ER) have been identified as a core mechanism in both NSSI (Andersson et al., 2022) and several mental disorders, including depression, anxiety, and eating disorders (Aldao et al., 2010). ER can be conceptualised as the processes through which individuals influence which emotions they have, when they experience them, and how these emotions are expressed and experienced (Gross, 1998). ER has also been described as a multidimensional construct involving abilities such as awareness, understanding, and acceptance of emotional responses, the capacity to engage in goal-directed behaviour while inhibiting impulsive actions, and the ability to modulate emotional responses while remaining willing to experience negative emotions in pursuit of meaningful activities (Gratz & Roemer, 2004). These abilities are reflected in several evidence-based therapeutic frameworks, including Dialectical Behaviour Therapy (Linehan, 1993) and Emotion Regulation Group Therapy (Gratz & Gunderson, 2006).

Adolescence is a sensitive period for the development of ER capacities (Steinberg, 2005), as ongoing cognitive, emotional, and social development influences how adolescents perceive, differentiate, and manage emotional experiences. Some ER-related capacities, such as emotion differentiation, have been shown to decrease during adolescence, potentially reflecting the increasing complexity and intensity of emotional experiences during this stage (Nook et al., 2018). At the same time, the social environment continues to play an important role in the development of ER. In particular, parents contribute to the development and maintenance of effective ER strategies in children and adolescents (Morris et al., 2017) and even into young adulthood (Xu et al., 2019). Parents’ own ER and emotion socialisation practices have been shown to influence how their offspring understand and regulate emotions (Xu et al., 2019). Accordingly, involving parents when addressing ER in adolescents may be particularly important during this developmental stage.

Several interventions that target ER have been developed for community and clinical samples of adolescents (Eadeh et al., 2021). Such examples include prevention-focused programmes generally and those addressing mental health problems, such as NSSI (Bjureberg et al., 2023). Interventions that target ER show promising results in reducing maladaptive ER in clinical and community samples, even though effect sizes in community samples are smaller (Eadeh et al., 2021). Holmqvist Larsson et al. (2020) have piloted an ER skills training group for adolescents and parents in a clinical setting with preliminary promising results. Participants experienced targeting emotions in this skills training as valuable and helpful (Holmqvist Larsson et al., 2023).

Regarding community-based prevention programmes, a variety of initiatives have been developed for adolescents, with school-based interventions representing one common and important form (Pedrini et al., 2022). School-based programmes may be primary or universal (i.e., delivered to all to prevent problems from occurring), secondary or selective (targeting adolescents at risk of developing mental disorders), or tertiary/indicated (preventing existing problems from getting worse). Overall, prevention programmes that target ER show promising results in reducing difficulties with ER and improving general health, mostly measured by self-reported difficulties in ER (e.g., emotional competence, goal-directed behaviour, and emotional awareness) and symptoms of anxiety and depression (Pedrini et al., 2022). This is confirmed by a meta-analysis that pointed out ER as one of three important components in prevention programmes (i.e., universally-delivered psychosocial interventions delivered to adolescents aged 10−19 years), as they were found to predict positive effects across several outcomes, such as increased mental health, as well as reduced anxiety and depressive symptomology (Skeen and Laurenzi, 2019).

Research on prevention programmes for parents of adolescents is scarce (Yap et al., 2016), and there is only a limited number of studies examining joint parent-adolescent preventive interventions targeting ER. Moreover, there are only a few qualitative studies exploring participants’ experiences of preventive ER interventions for adolescents in a school setting (Burckhardt et al., 2017; Lakes and Nguyen, 2019). There is a need to specifically explore the perspective of both parents and adolescents participating together. While available studies report mostly positive outcomes, such as increased emotional competence and greater understanding and expression of emotions among adolescents (Lakes and Nguyen, 2019), this gap means that it is unclear how joint participation shapes parents’ and adolescents’ experiences, perceptions of ER skills, and their application in daily life; insights critical for refining school-based prevention to strengthen family-level support for adolescent mental health.

In addition to individual-level factors, broader societal influences, such as the impact of digital media, have been suggested to contribute to emotional stressors and the rise in mental health problems among adolescents (Haidt, 2024). As such, addressing adolescent mental health requires not only individual-level interventions but also structural changes that target the societal conditions contributing to these challenges.

The field of prevention science is expanding, and there is an ongoing discussion regarding which aspects to consider when conducting preventive interventions for mental health problems in adolescence. Some important aspects are, for example, who should be targeted, and at what level of risk different preventive interventions are most effective. The issue of who could potentially benefit must also be weighed against the cost and effectiveness of the intervention when considering what level of prevention to choose. Other aspects to consider are timing, in which context preventive interventions are best delivered, and what precursors to address, such as emotion dysregulation, self-criticism, or communication (Heath et al., 2014). Given the limited research on how adolescents and parents experience joint, school-based preventive ER interventions, this study aims to explore adolescents’ and parents’ experiences of participating in a preventive ER skills training delivered in a school setting. The study focused on the following research questions:

- How did adolescents and parents describe their experiences of participating in the ER skills training group?
- How did participants perceive addressing emotions and ER in a joint, school-based setting, including perceptions of benefits and challenges?

## Method

### Design

The current study explores the experiences and perceptions of adolescents and parents who participated in an ER skills training in a school setting. The intervention was embedded within a broader school-based prevention programme targeting NSSI and mental health (Aspeqvist et al., 2024, 2025), which comprised interventions delivered to adolescents, parents, and school staff. The present study specifically examined participants’ experiences of the ER skills training as one component of this larger programme. Both adolescents and their parents were invited to participate in a joint ER skills training conducted in the school setting after regular school hours.

### Participants and procedure

The ER skills training was offered to adolescents (13−15 years in grades 7 and 8) and parents participating in the larger prevention programme (Aspeqvist et al., 2025). Adolescents and both caregivers provided written informed consent in the larger project, which included the possibility of participating in the ER skills training. Participants received both verbal and written information about the skills training, including the purpose, confidentiality, and the voluntary nature of participation. Information and an invitation to enrol in the ER skills training were provided during school-based parent meetings and via email to parents who had given informed consent to participate in the broader prevention programme. The ER skills training consisted of five sessions, á two hours, which was piloted previously in clinical groups (Holmqvist Larsson et al., 2020). The skills training was voluntary for both adolescents and parents, and the participants could withdraw at any time without any consequences. Licensed psychologists with clinical experience in ER skills training and adolescent mental health conducted the training. Additionally, participants were informed about where to seek further support if they were to experience any adverse effects. See Table I for an overview of the content of the ER skills training and supplementary materials for a more detailed description. Two separate ER skills training groups were conducted for a total of seven families. One skills training was held at a central location in May-June 2022 with two families (two adolescents and two parents). The other group was held at the school premises in November-December 2022 with five families (five adolescents and six parents). One family dropped out after one session due to logistical difficulties.

**Table I.** Overview of the Content of the Emotion Regulation Skills Training.

Sessions	Content
Session 1	Awareness of emotions and labelling emotions
Session 2	Identifying emotions and the functions of emotions
Session 3	Primary and secondary emotions. Validation and reducing judgement
Session 4	Reducing vulnerability and emotional imbalance
Session 5	Making conscious choices—goal-directed behaviours

No additional inclusion or exclusion criteria were applied for participation in the ER skills training beyond enrolment in the broader school-based prevention programme (Aspeqvist et al., 2025), which was delivered universally to all students in grades 7 and 8 in regular school classes (i.e., following the standard Swedish curriculum) at participating schools. Thus, participation in the ER skills training was voluntary, and families self-selected into the ER skills training.

All participants were invited to take part in an interview about their perceptions and experiences of participating in the skills training. One adolescent and the family that dropped out did not want to participate in the interview, resulting in seven parents (two fathers and five mothers), and five adolescents, aged 13–15 years (with equal gender distribution), being interviewed. The study was approved by the Swedish Ethical Review Board (2021–01699, 2021–05049).

### Data collection

The interviews were held approximately two weeks after the skills training was completed (participants of the first skills training group were interviewed in June 2022, and the second group was interviewed from December 2022—January 2023). HA interviewed the participants of the first group, and EA and a research assistant interviewed the second group of participants. HA was a skills trainer in the second group and consequently did not interview those participants. All interviewers were licensed psychologists with previous clinical experience working with children and adolescents in different settings. The participants were interviewed separately via Zoom. At the beginning of the interview, the participants were reminded of the study's purpose, confidentiality, and their right to withdraw at any time.

The interviews lasted between 14 and 49 minutes, with an average of 33 minutes. The interview was semi-structured with open-ended questions to capture diverse perceptions and experiences of participating in the ER skills training, see interview guide in supplementary materials. Interviews were audio-recorded and transcribed verbatim.

### Data analysis

The analysis aimed to interpret patterns of shared meaning across the dataset, focusing on participants’ experiences and perceptions of the skills training. Reflexive thematic analysis was chosen given the open-ended research question, as we aimed to generate themes that captured patterns and variations in participants' accounts. The analysis was mainly inductive and grounded in the data, engaging with both semantic and latent levels of meaning, while remaining close to participants’ descriptions (Braun & Clarke, 2023). See supplementary material for an example of the analysis process of the relationship between overarching themes, subthemes, codes, and meaning units. A critical realist approach was used in the study, which assumes that there is a reality independent of the researcher (Fletcher, 2017). The knowledge of reality is, however, created by social structures (Fletcher, 2017). In this study, we thus acknowledged the influence of contextual factors on both the participants' descriptions and our interpretations of their experiences in the ER skills training. However, from this point onwards, participants’ descriptions of their experiences are also referred to as experiences. The aim was to generate themes of how the ER skills training was perceived.

Transcripts were analysed by HA, MZ, and EA using the six steps in reflexive thematic analysis (Braun & Clarke, 2006, 2019, 2022). Coding and theme development were conducted collaboratively, with analytic decisions discussed and refined through team discussions (see supplementary materials for a detailed description of the analysis process). HA, MZ, and EA are all licensed psychologists with experience working with children, adolescents, and their parents in different settings (schools, child and adolescent psychiatry, and primary care), with methods such as CBT, ACT, and DBT. BMG and LK have extensive experience in child and adolescent psychiatry, preventive work, and qualitative research. HA and MZ were also skill trainers of the ER skills training group delivered in the current study. HA, BMG, LK, and MZ are females, and EA is male.

Reflexivity was an ongoing consideration throughout the research process. Within a reflexive thematic analysis framework, researcher subjectivity is understood as shaping, rather than biasing, knowledge production. The researchers’ clinical experience with ER-based interventions and involvement in the skills training could have influenced how participants’ accounts were interpreted, for example, by sensitising the researchers to particular aspects of the intervention or creating expectations regarding its perceived usefulness. To enhance transparency and critical reflection, interviewers did not interview participants from groups in which they had served as skills trainers. The analysis was conducted collaboratively, with regular discussions among authors who were and were not part of the intervention to challenge interpretations, reflect on preconceptions, and consider alternative understandings of the data. The diversity of professional experiences within the research team also enabled different perspectives to be brought into the analytic process, supporting a more critical and reflexive engagement with the data.

## Results

Three overarching themes were generated in the analysis: *A hidden need for emotional awareness, The social context as a catalyst for emotion regulation,* and *Gains and challenges.* Each theme and subtheme is presented in Table II.

**Table II.** Overarching Themes and Subthemes.

Overarching themes	Subthemes
A hidden need for emotional awareness	A blind spot Silenced by stigma
The social context as a catalyst for emotion regulation	Seeing oneself through others Parents as facilitators Creating a safe environment
Gains and challenges	Insights Difficulties evaluating outcomes

### A hidden need for emotional awareness

The overarching theme *A hidden need for emotional awareness* describes a perceived need for increased knowledge of emotions in adolescents and parents. Here, emotional awareness includes knowledge of emotions and ER, as well as awareness of one’s own emotions and the role of emotions in participants’ everyday lives. The need for emotional awareness was interpreted as hidden, as participants expressed being unaware of the need for ER skills in everyday life and experiencing stigma related to emotional expression.

#### A blind spot

A majority of participants, including parents and adolescents, described experiencing a lack of information and education about emotions, what ER is, and how knowledge about emotions can be helpful, prior to participating in the preventive ER skills training. They also expressed being unaware of lacking this knowledge (that is, before the training, they had not recognised this gap), and that they had not previously considered the role of emotions and ER in the conceptualisation of problems they encountered in everyday life. This lack of knowledge was also pointed out as a barrier to enroling in the ER skills training group, as participants initially did not understand what ER was, and in which way it could be useful for them.

I also think that you don’t really know fully what it means … because a colleague I spoke to didn’t really know what it was and because we had already participated in it [ER skills training] before, so we knew what emotion regulation meant, but I think there’s a lot of people not really, like understanding what it is and how helpful it is. [Parent 5]

Exemplifying this further, some parents described a general lack of knowledge about primary and secondary emotions and the potential negative consequences of not being aware of your emotions. For instance, parent 3 said: “I think that there’s a lot of people out there that don’t have the knowledge about this. It’s so easy to get angry, yeah, but what’s behind that anger?” Parent 1 compared the lack of knowledge of emotions and ER to education in other school subjects and receiving information about physical but not mental health:

…but you really don’t do that [talk about emotions or mental health problems], you learn about various viruses and anatomy and biology and sexual science and all of those various things, right? But you don’t get into the mental parts with the adolescents.

This knowledge gap was also expressed as a reason for the uninterest prior to enroling in the ER skills training among the adolescents. Some of the adolescents said that they initially thought they already had sufficient knowledge about emotions and ER, but after participating, realised that they had previously been unaware of this knowledge gap.

Taken together, the initial lack of knowledge about emotions and ER and a prior lack of awareness of this knowledge gap were conceptualised as *A blind spot*.

#### Silenced by stigma

The participants, adolescents as well as parents, expressed concerns about being stigmatised when participating in a group with a focus on mental health in general and emotions specifically, as well as fears of talking about emotions in different settings. They perceived that it was important to talk about emotions, but that this, however, was not done regularly. The general perception was that teenage boys were especially reluctant to talk about emotions, and Adolescent 4 expressed that difficulties in talking about emotions among boys were related to gender norms in school:

Because like now it’s quite crazy and like if a guy is feeling an emotion, it’s wrong. Then the reaction is like “haha you want to cry haha”. Like the whole school, all guys in school shouldn’t show any emotions.

Participants interpreted the absence of talking about emotions or mental health in general as problematic, which they thought could lead to nonacceptance of negative emotions. Adolescent 2 described that talking about emotions could make it more acceptable:

[There were] a lot of new things [in the ER skills training] because you don’t talk about emotions in school, not much at all, and then it feels like you don’t talk about feeling bad, and then it feels like you’re not allowed to feel bad. Everyone should get to know that it’s okay to feel bad, it’s, like, okay.

Social fears, concerns about how to be perceived, a fear of being judged, or the risk of being perceived as having mental health problems were further discussed as barriers to enroling in the ER skills training and talking about emotions. Taken together, perceived stigma surrounding mental health problems and emotional expression was interpreted as obscuring the need for emotional awareness among the participants.

### The social context as a catalyst for emotion regulation

Adolescents and parents perceived the social context in the preventive ER skills training as important. The social context was interpreted as being a catalyst for ER, as it was expressed as potentially hindering or facilitating learning and using ER skills, and emotional expression.

#### Seeing oneself through others

There was a general agreement between adolescents and parents concerning the positive effect of listening to others talk about their emotional experiences in the group setting. The possibility of receiving a perspective of one’s situation by listening to others affected how the participants experienced their situation. Listening to others generated positive feelings related to being validated, and experiences of not being alone, as well as recognising oneself in others.

It was like a bit of a relief because when they brought up… how their family functioned, you recognised yourself, like yeah that’s how we do sometimes as well and it was a bit of a relief to think that it wasn’t just us doing that. [Adolescent 1]

The central aspect of listening to others was the normalisation of emotions and being able to relate to others. Adolescent 1 expressed that she was hesitant initially to talk about her emotions in the ER skills training group, but when she did so received help from the skills trainers, who made her experiences general and relatable to others, which was appreciated. Experiences of not being able to relate to others or to the examples that were discussed were described more negatively.

#### Parents as facilitators

Adolescents generally described the presence of parents as supportive, and no contrasting or negative accounts of parental involvement emerged in the data. Parents made them feel safe, helped them understand the material, were role models, and helped them generalise their knowledge. The parents were interpreted as being facilitators in the social context of the skills training, making it easier for the adolescents to express emotions and practice ER skills. All adolescents described a sense of safety in having their parents with them in a group of new people. This sense of safety appeared to facilitate adolescents’ engagement in the group and their willingness to participate. Adolescent 5 said “Yeah, it felt a bit safer [participating together with a parent]. If there had been a group where I didn’t know anybody, I would probably be much less social.” In addition, parents could support adolescents’ understanding of the material by providing examples and sharing their own emotions and experiences, which appeared to help adolescents engage with and make sense of the skills introduced in the training. Adolescent 1 expressed the value of having parents in the ER skills training group: “I think it was the adults in the group that maybe understood a bit better who created discussions that helped us adolescents to understand a bit better what we were talking about.” Some participants also mentioned that parents could help their adolescents practice the skills in everyday life, generalising skills by talking about them at home, and helping adolescents with their homework assignments.

#### Creating a safe environment

The participants talked about their perceptions of being in a group, the group climate, and specifically discussing emotions in a new group constellation. The environment was interpreted as influencing whether participants felt safe expressing their emotions. Feelings of safety appeared to facilitate engagement in the skills training, which in turn could create conditions that supported learning and practicing skills. The experiences varied. Most participants experienced that it was easy to talk and share experiences in the group. However, a few parents experienced that their adolescents were hesitant to talk in front of others. Parent 1 thought that the sense of feeling safe in the group grew during the ER skills training.

… I also felt that it took a couple of times before you felt safe in the group and before everybody could participate in the discussions... and it was actually only at the last session that the adolescents joined the discussions. So, I absolutely think it would have been good to have a couple of more sessions.

### Gains and challenges

The overarching theme *Gains and challenges* describes participants’ perceptions of perceived outcomes of the ER skills training in a preventive context and challenges related to these gains.

#### Insights

After participating in the ER skills training in a school setting, both adolescents and parents described gaining new knowledge about emotions and ER and emphasised that such knowledge is important for everyone. This subtheme was labelled *Insights* to capture participants’ descriptions of realising or becoming aware of aspects of emotions and ER that they previously had not recognised. These insights were reflected in participants’ description of increased awareness of emotions, new ways of interpreting others’ behaviour, and reflections on how ER skills could be applied in everyday situations. Participants described increased awareness and understanding of their own and others’ emotions and reflected on how ER skills could be useful in different situations, particularly in more challenging situations in life.

But I think most of those participating…became more aware of themselves and others too.. you notice a bit like yeah now that person did that and it might not have been what that person meant, he/she might just have been angry.. you’re a bit more, you see what people feel even though they don’t like say it. [Adolescent 1]

The parents also expressed a need to keep on practicing the skills they had learned, and some parents made a distinction between increased awareness of emotions and actual behavioural change. Parent 5 described having become more aware of emotions, but still needing to practice:

Yeah, I think so, and we discussed that a lot, sure you realise it afterward and maybe you do that in the beginning before you learn how to use them [the ER skills] fully and then you think that yeah maybe it’s better to think about it and realise it afterward than not at all, maybe you get closer each time.

The insights were also discussed in relation to the timing of the intervention. Some of the parents stressed the need to receive this information earlier, especially when their adolescents were younger, and during times in the adolescents’ development with more conflicts in the family. Parent 5 said, “As a parent, you would like to have this [ER skills training] earlier, as I said before, because it’s often more conflicts when they’re younger maybe, and you can get a solid ground to stand on.”

Parents also considered that the adolescents needed this information and discussed the timing of the ER skills training, weighing the potential benefit of receiving ER skills earlier against whether adolescents would be ready to engage with the material at that age. Adolescents expressed a change of opinion related to the gained insights. Initially, they did not see the need for the ER skills training or were less interested in participating, but afterward, their perspectives changed, and they said that all adolescents would benefit from this knowledge.

#### Difficulties evaluating outcomes

Some participants expressed difficulties evaluating the effects of the ER skills training. As the intervention was delivered preventively, the participants did not experience a great burden of problems prior to participating. Most of the participants described having gained knowledge and insights, but did not experience a great effect on the level of problems or symptoms. This was conceptualised as difficulties evaluating outcomes, as participants often described having relatively few problems prior to the intervention and therefore found it difficult to assess changes. Parent 4, for example, expressed difficulties evaluating if the family relationships had been affected because they did not experience relational problems in the family before the ER skills training:

It’s hard to say, I think that it hasn’t done anything worse at least, but I wouldn’t say that it has any, yeah…we didn’t get into this because of any big problems.

## Discussion

The main finding of this study was that the participants, after having taken part in the ER skills training, identified a need for emotional awareness and knowledge about emotions that they had not previously been aware of. Unawareness of the role of emotions in the conceptualisation of problems encountered in everyday life, and perceived stigma surrounding mental health problems and emotional expressions obscured the need for knowledge about emotions and ER. Further, the social context was interpreted as a catalyst for ER, hindering or facilitating participants’ emotional expressions, the use of ER skills, and learning ER skills. The participants experienced several meaningful outcomes of the preventive ER skills training, such as increased awareness and knowledge of emotions, and gained ER skills, interpreted as Insights. The meaningful outcomes were furthermore associated with challenges, for instance, concerning the timing of the intervention and outcome measurements.

### A hidden need for emotional awareness

Adolescents and parents appreciated the knowledge gained and considered it generally valuable. Participants did not report harm from increased emotional awareness but noted that stigma and lack of knowledge had previously obscured the importance of emotions and ER skills. This aligns with prior research on universal ER interventions improving mental health and reducing symptoms of depression and anxiety (Skeen and Laurenzi, 2019) and confirms that preventive ER training is perceived as meaningful. In young adults, emotion self-stigma (i.e., believing that it is unacceptable to experience or express negative emotions) has been associated with lower levels of self-rated likelihood of seeking help from formal and informal sources (Harvey & White, 2023). The perceived stigma that participants experienced surrounding mental health and talking about emotions could therefore be a barrier to identifying adolescents at risk, as they may not seek help. Emotional awareness, here defined as the perceived ability to identify and express one’s emotions to others, has further been associated with an increased likelihood of self-rated help-seeking in a sample of community adolescents as well as in young adults (Harvey & White, 2023). Thus, targeting emotions and ER at a primary/universal level could potentially be beneficial to increase help-seeking in adolescents in need of help. Stigma and unawareness were also interpreted as barriers to enroling in the ER skills training, which confirms previous research (Finan et al., 2019) that has explored reasons not to enrol in prevention interventions in a group of non-engaged parents. For instance, a perceived lack of initial problems (and perceiving not being affected by mental health problems), concerns about being stigmatised, discussing sensitive or private topics, adolescents not wanting to or not seeing a need for the knowledge and unclear advertisements were expressed by parents as potential barriers to enrol in a prevention programme according to the findings of Finan and colleagues in their study in Australia, where most of the preventive programmes were delivered in a school setting (Finan et al., 2019). Several of these aspects were confirmed by participants in the current study. Other obstructing factors identified by Finan and colleagues were transportation, childcare, and concerns about whose responsibility it is to prevent and educate about mental health issues. These potential barriers were not, however, expressed by participants in the current study. This may be a result of the current study only exploring the perceptions of the parents who enroled, who may not have experienced troubles with transportation or childcare, for instance.

### The social context as a catalyst for emotion regulation

The social context influenced participants’ ER, with group participation offering validation, support, and a sense of not being alone, mechanisms previously observed in clinical DBT and ER training samples in group settings (Holmqvist Larsson et al., 2023; Ohlis et al., 2023). These mechanisms were similarly reported in our community sample, suggesting the potential benefit of group-based preventive settings.

Research regarding universal, selective, or indicated prevention of internalising problems for parents of adolescents is scarce (Yap et al., 2016), and the results of the current study highlight several advantages of including parents when working with ER skills training preventively for adolescents. Adolescents described feeling safer participating together with their parents, and that parents facilitated adolescents’ knowledge acquisition and generalisation of skills. This could be especially important in learning ER skills, as parents play an important role in the socialisation of children’s and adolescents’ emotions (Morris et al., 2017; Xu et al., 2019). However, practical concerns regarding involving parents in preventive interventions in school settings need to be addressed. Parental non-engagement, including difficulties recruiting parents, is one such concern (Finan et al., 2019). The generally positive feedback from adolescents regarding the inclusion of parents in the intervention may be influenced by the characteristics of the current sample. Families with high levels of conflict, for example, could be less inclined to sign up for a joint intervention. It is important to acknowledge that the potential benefits of involving parents in the treatment of children and adolescents may vary depending on several factors, including the nature of the parental involvement (Lawrence et al., 2021) and the specific issues being addressed (Pine et al., 2024). Some adolescents may perceive parental involvement as intrusive or obstructive (Harper et al., 2014). This highlights the need for further research in this area, particularly in the context of preventive interventions, to better understand the effects of parental involvement.

The social context was also perceived by participants as a potential barrier to expressing emotions, and the importance of creating a safe environment needs to be considered when delivering ER skills training to prevent mental health problems among adolescents. The importance of the social context for ER has been highlighted in previous studies (English et al., 2017; Gross & Cassidy, 2019; Wylie et al., 2023). In the present study, participants also described gender norms within the school environment that discouraged boys from expressing certain emotions, suggesting that social expectations around masculinity may limit adolescents’ willingness to openly discuss emotional experiences. Traditional masculine norms may discourage certain emotional expressions and help-seeking among boys and young men (Addis & Mahalik, 2003; Seidler et al., 2016), and the stigma surrounding mental health problems can act as a barrier to help-seeking in adolescents (Gulliver et al., 2010). Such norms may influence both engagement in emotion-focused interventions and adolescents' opportunities to practice ER skills in their everyday social environments. These findings highlight the potential value of preventive, school-based interventions that normalise emotional expression and create spaces where adolescents can discuss emotions without fear of negative peer reactions.

### Gains and challenges

Participants in the current study reported becoming more aware of and better equipped to regulate emotions, consistent with prior school-based preventive programmes in which children gained emotional awareness and adolescents improved understanding and expression of emotions (Lakes and Nguyen, 2019; Skryabina et al., 2016). These programmes were not, however, delivered jointly to adolescents and parents. Even though the participants in the current study described that they had learned several skills and gained valuable insights, they also experienced a continued need to keep on practicing, and that they were only at the beginning of using skills in their everyday lives. The acquisition of knowledge, increased emotional awareness, and emerging skills can therefore be considered meaningful outcomes in themselves, independent of changes in symptoms or problem behaviours. How to maintain skills is one aspect that needs special attention when teaching ER skills, in combination with the need for contextual and structural changes that reinforce the use of healthy ER skills. Participants also discussed the timing of the intervention, and some related the need for the intervention to times in life when they were experiencing more problems requiring increased ER strategies. Heath et al. (2014) point out the importance of timing for prevention interventions and specifically emphasise the importance of conducting the intervention right before the development of the behaviour that one wants to target. In terms of ER and the development of ER difficulties, there seem to be different sensitive periods, where adolescence is one (Nook et al., 2018; Steinberg, 2005). This corresponds to the participants’ experiences of early adolescence being an important time to intervene preventively with the teaching of ER skills. However, for some parents, there seemed to be a need for ER skills at an earlier stage in the child’s development.

The school context may also be particularly relevant when considering developmental readiness. Early adolescence is a period characterised by rapid emotional, cognitive, and social changes, during which young people increasingly encounter complex emotional and interpersonal situations within school environments (Casey, 2015; Sawyer et al., 2018). Because most adolescents spend a large portion of their time in school, it provides a natural and timely setting to introduce ER skills. Delivering ER training in this context may therefore support adolescents at a stage when emotional demands are increasing, while still being early enough to foster the development of adaptive ER strategies.

Participants found it challenging to evaluate outcomes in terms of symptom reduction due to low pre-existing mental health problems, which illustrates a common floor effect in preventive research (Eadeh et al., 2021). Participants frequently reflected on outcomes in terms of reductions in problems or symptoms, which may reflect how psychological interventions are commonly understood, where improvement is often framed as symptom reduction. It is therefore possible that the phrasing of interview questions or how the intervention was presented influenced how participants conceptualised outcomes, potentially making changes related to increased awareness or skills less salient. Qualitative methods capture meaningful experiential outcomes—knowledge, skills, and personal growth—that are central to primary prevention and often overlooked by quantitative measures. Similarly, Burckhardt et al. (2017) found that adolescents participating in a school-based, emotion-focused preventive intervention reported mostly positive experiences, even though quantitative measures showed no significant differences between the intervention and control groups. This highlights why qualitative and experiential measures are particularly valuable in capturing meaningful outcomes that go beyond symptom reduction, even if such effects were not directly measured in the current study.

### Implications and further research

#### Implications for practice

The participants’ experiences explored in the current study have implications that need to be considered when conducting ER skills training in a preventive context. First, adolescents and parents experienced ER skills training as meaningful and perceived that it resulted in both increased knowledge about emotions and emotional awareness, as well as gained ER skills. It thus seems acceptable for adolescents and parents to participate together and work with emotions and ER preventively in a school setting. However, only 11 out of 267 eligible families showed an initial interest in participating. Results from the current study indicate that participants’ experiences of stigma related to participating in ER skills training and expressing their emotions, as well as a lack of knowledge about the role of emotions in everyday life, could be potential barriers to enroling. Such barriers, therefore, need to be addressed. Recruitment procedures and the framing of the intervention are critical considerations. Information about the ER skills training should be communicated clearly and accessibly, ensuring that potential participants understand the content of the programme as well as how the skills may be relevant and potentially useful in everyday situations. Involving parents and adolescents in this process is essential. While participants in the current study reported positive experiences with ER skills training, considering that it could be valuable as a universal intervention, several concerns warrant careful consideration. The effectiveness of universal prevention programmes varies (Tanner-Smith et al., 2018), and universal interventions may not be equally beneficial for all individuals (Spilt et al., 2013). Consequently, the cost-effectiveness of universal interventions must be carefully evaluated. Additionally, recent findings have highlighted the negative effects of a universal preventive programme based on DBT skills (Harvey et al., 2023), underscoring the need for further research in this area.

#### Implications for policy

Adolescent mental health is influenced by a complex interplay of factors beyond individual ER. Bullying (Li et al., 2022), for example, is a significant contributor to mental health issues, and determining which factors should be prioritised in prevention efforts remains unclear. Importantly, mental health challenges are not merely individual problems but are embedded within broader systemic and societal contexts that also require attention. Haidt (2024), for instance, argues that modifications to the environments in which children and adolescents develop are necessary, particularly in relation to digital media use and school contexts. Such environmental and systemic interventions may include school policies that promote structured smartphone use or screen-free time during the school day, anti-bullying and inclusion initiatives, teacher training in social–emotional learning, and the creation of psychologically safe classroom climates. At a broader societal level, this may also involve parent-focused programmes, public health campaigns aimed at reducing stigma around mental health, and policies that support family well-being, such as accessible mental health services. These considerations suggest that a more comprehensive and multifaceted approach to mental health prevention is warranted. Individual-level interventions, such as ER skills training, may strengthen adolescents’ capacity to manage emotional challenges, while systemic and environmental changes can reduce chronic stressors and create contexts that support the application of these skills. Integrating individual-focused programmes with school-wide, family-level, and societal initiatives may therefore enhance both the sustainability and overall impact of prevention efforts.

#### Implications for research

Future research should consider what to include as outcome measurements when evaluating prevention interventions generally, and ER skills training specifically. For instance, the need for measuring skills and strengths as outcomes in prevention has been discussed in previous research (Eadeh et al., 2021), and the experiences of the participants in the current study also emphasise the importance of focusing on aspects such as strengths and skills gained, rather than symptom reduction. Examining the sustainability of learned ER skills over time would also be valuable. Longer-term follow-up assessments (e.g., 3–6 months post-intervention) could provide insight into how these skills are maintained and integrated into everyday life. Future research should also explore the perspectives of individuals who discontinue participation in similar interventions, as this could provide a more comprehensive understanding of barriers to engagement and programme acceptability. Furthermore, more research regarding preventive interventions for parents of adolescents is needed (Yap et al., 2016). The results of the current study point to experiences of positive effects of conducting prevention interventions for adolescents and parents jointly. As adolescents spend much of their time in school, the school context may represent an important setting for supporting the development of ER skills. Although the present study focused on adolescents and parents, teachers influence classroom emotional climates and may therefore represent a potential avenue for support. While teacher involvement was not examined here, some research suggests that mental health literacy training may improve teachers’ knowledge and confidence in responding to students’ mental health needs (Imran et al., 2022). Future research could explore whether teacher-focused components might complement student- and parent-focused ER interventions in school settings.

Furthermore, the intervention consisted of five sessions, reflecting a pragmatic balance between exposure to core skills and feasibility in a voluntary school-based setting. A longer format may provide greater opportunity for skill consolidation. Future studies should examine whether extending the intervention influences participants’ perception of maintenance of ER skills and longer-term outcomes.

## Limitations

There are some limitations to consider when interpreting the results. First, since only a few parents and adolescents signed up for the ER skills training, this likely influenced who chose to participate. This may lead to potential bias toward motivated or emotionally aware families, which may affect the transferability of findings. The recruited participants may have had a greater interest in emotions and ER, or may have been in greater need of the intervention, for example. Low socioeconomic status and limited social support are some examples that have previously been identified as barriers to enroling in preventive interventions (Baker et al., 2011), as well as low levels of parental reports of adolescent symptomatology and problems (Finan et al., 2018). Information on the current sample demographics for these variables is lacking, particularly with regard to socioeconomic, cultural, and educational background characteristics among those who enroled in the ER skills training. This limits our ability to assess whether participation and outcomes may have differed across demographic groups and therefore constrains the contextual interpretation and transferability of the findings. However, factors such as interest in the subject and perception of the parent-child relationship could also be factors that influence the choice of enroling. Additionally, the two ER skills training groups were conducted at different time points and in different settings, which may have introduced contextual influences on participants’ experiences (e.g., seasonal factors, familiarity with the school environment, or group dynamics). However, no notable differences in participants’ accounts were observed between the two groups in the analysis. Future studies could nevertheless consider more systematically examining how contextual factors across intervention groups may shape participants’ experiences. The study relied exclusively on participants’ narratives, which is consistent with the reflexive thematic analysis approach and the aim of exploring subjective experiences rather than establishing objective accounts. Nevertheless, incorporating additional perspectives (e.g., teachers, peers, or independent observers) in future research could provide complementary insights and contribute to a broader understanding of how the intervention is experienced and enacted across contexts.

Lastly, it was decided to analyse the transcripts of the adolescents and the parents together, as the answers were similar and surrounded the same themes of interest. Analysing them separately could yield different themes not captured in the current study. However, all perspectives, including those unique to parents or adolescents, were analysed with equal weight and were not marginalised or excluded based on their frequency.

## Conclusions

This study is the first to explore adolescents’ and parents’ experiences of participating in a joint ER skills training group delivered preventively in a school setting. The results highlight participants’ experiences of important issues currently discussed in preventive research, including who should be targeted, the timing and context of interventions, and the focus of the programme. Participants found the training valuable and meaningful, reporting increased emotional awareness and knowledge of ER strategies, needs that may be obscured by stigma or lack of prior knowledge, and identified the social context as both facilitating and hindering the use and learning of ER skills. By capturing these experiential insights, the study provides a unique qualitative contribution to ER research, revealing processes and needs not easily detected by quantitative methods. The findings have potential to advance theory by emphasising the developmental and social dimensions of ER, inform practice by highlighting considerations for intervention timing, target groups, and parental involvement, and suggest methodological refinements for preventive programmes, including broadening outcome measures beyond symptom reduction to encompass knowledge, skills, and personal growth.

## Supplementary Material

Supplementary Material ZQHW-S-2025-0605.R2-Supplementary.docx

## Data Availability

The data that support the findings of this study cannot be made publicly available for confidentiality reasons. Data are however available from the corresponding author upon reasonable request subject to ethical permissions and participant consent.
