# Supplementary material for: Everyone should get to know that it’s okay to feel bad: adolescents’ and parents’ experiences of participating in a preventive emotion regulation skills training in a Swedish school setting — a qualitative study
Source: Int J Qual Stud Health Well-being. 2026 May 23;21(1):2679416. doi: 10.1080/17482631.2026.2679416 (PMC13202694; doi:10.1080/17482631.2026.2679416)
Supplement: Supplementary Material — ZQHW-S-2025-0605.R2-Supplementary.docx [file ZQHW_A_2679416_SM2937.docx]

**Supplement Material**

Everyone should get to know that it’s okay to feel bad: Adolescents’ and parents’ experiences of participating in a preventive emotion regulation skills training in a Swedish school setting — A qualitative study

**Description of ER Skills Training**

The skills training was inspired by evidence-based Cognitive-Behavioral Therapy treatments with ER as a core component, such as Dialectical Behavior Therapy (Linehan, 1993), Emotion Regulation Group Therapy (Gratz & Gunderson, 2006), Acceptance and Commitment Therapy (Hayes, 2012), and Unified Protocol (Barlow, 2011), as well as by child and adolescent psychiatric practice and guidelines.

The overall aim of the skills training was to increase emotional awareness and reduce difficulties with emotion regulation. The content included psychoeducation with information on what emotions are, skills for identifying, labeling, and expressing emotions, validation, and acceptance, as well as reducing emotional vulnerability. Participants received homework assignments between the sessions, which were followed up and discussed jointly in the group.

**Description of Data Analysis**

[Anonymized] transcribed the interviews carried out by [Anonymized] and a research assistant as part of the familiarization process. The interviews carried out by [Anonymized] were transcribed by an assistant in the research group. [Anonymized] and [Anonymized] analyzed three transcripts individually and together to explore differences regarding meaning units, codes, and themes. Thereafter, [Anonymized] analyzed the rest of the transcripts. [Anonymized] gave input in earlier stages of the analysis and suggested themes. The transcripts were read and re-read, coded, and themed, in a process moving back and forth between these steps, altering codes and themes after checking if the themes worked in relation to the codes and transcripts, but also discussing the generated themes and separating/combining themes to capture the shared and underlying meaning. The analysis was discussed and reviewed with [Anonymized] throughout the process. [Anonymized] labeled the themes, and all authors discussed and approved the results.

**Example of the Analysis Process: the Relationship Between Overarching Theme, Subthemes, Codes, and Meaning Units**

| Overarching theme | The social context as a catalyst for emotion regulation | | |
| --- | --- | --- | --- |
| Subtheme | Seeing oneself through others | Parents as facilitators | Creating a safe environment |
| Codes | - Getting new perspectives - Normalizing - Recognizing oneself, not experiencing oneself as alone | - Parents make adolescents feel safe - Parents help with generalizing knowledge - Parents help with understanding the material | - Concerns about speaking in front of others - The safety in the group is important - The size of the group matters - Who participates in the group - Fears of knowing someone in the group |
| Meaning units | “It was like a bit of a relief because when they brought up… how their family functioned you recognized yourself, like yeah that’s how we do sometimes as well and it was a bit of a relief to think that it wasn’t just us doing that” | “Yeah, it felt a bit safer [participating together with a parent]. If there had been a group where I didn’t know anybody, I would probably be much less social” | “… I also felt that it took a couple of times before you felt safe in the group and before everybody could participate in the discussions... and it was actually only at the last session that the adolescents joined the discussions. So, I absolutely think it would have been good to have a couple of sessions more.” |

**Interview Guide (Translated from Swedish)**

Can you tell me how it came about that you decided to participate in the emotion regulation skills training group? Was there anything in particular that made you interested or led you to sign up?

Was there anything that made you hesitant about participating?

Were there any potential barriers for you to participate? What kinds of things might have made it difficult for you to take part?

How would you describe your ability to manage emotions before participating in the group?

What are your thoughts about being offered this emotion regulation skills training preventively at school (rather than, for example, through mental health services)?

How was it to work with emotions and learn about emotions in a preventive context?

What do you think about the timing of being offered this emotion regulation skills training in grade 7/8? Do you think it would have been better earlier or later?

Who do you think would benefit from participating in this group? In what way?

How do you think more people could be reached with this type of emotion regulation skills training?

What do you think might be the biggest barrier for others when it comes to signing up?

Was there anything about the group that you experienced as less helpful or less positive? For example, aspects related to it being delivered in a school setting or as a preventive intervention.

Can you tell me about your experience of participating in the group?

What do you think was the most important lesson or knowledge you gained from participating in the group?

What did you find most helpful?

Was there any content that you found particularly difficult to understand, apply, or practice?

Have you continued to use any of the emotion regulation skills in your everyday life?

Do you think that participating in the group has affected you in any way? If so, how?

How did you experience participating together with other adolescents/other families?

How was it to participate in the group together with your parent/your child?

Do you feel that participating in the group has influenced how you talk to each other within the family?

Do you think it has affected the relationship between you and your parent/your child?

Do you notice any changes in your parent/your child as a result of participating in the group?

Do you think others notice any difference in you after participating in the group?

If I asked you about this again in a year, what do you think you would remember? What might be different?

What did you think about the format and structure of the emotion regulation skills training, such as watching PowerPoint presentations, receiving handouts, watching videos, completing homework assignments, and meeting for two hours once a week?

Is there anything you think could be done differently? For example, something that could be removed, added, or changed?

Is there anything else you would like to add?

# **References**

Barlow, D., Farchione, TJ., Fairholme, CP., Ellard, KK., Boisseau, CL., Allen, LB., et al. (2011). *Unified protocol for transdiagnostic treatment of emotional disorders: Therapist guide.* Oxford University Press.

Gratz, K. L., & Gunderson, J. G. (2006). Preliminary data on an acceptance-based emotion regulation group intervention for deliberate self-harm among women with borderline personality disorder. *Behav Ther*, *37*(1), 25-35. <https://doi.org/10.1016/j.beth.2005.03.002>

Hayes, S., Pistorello, J., Levin, ME. (2012). Acceptance and Commitment Therapy as a Unified Model of Behavior Change. *The Counseling Psychologist.*, *40*(7), 976-1002.

Linehan, M. (1993). *Cognitive-behavioral treatment of borderline personality disorder*. Guilford Press.
